# Supplementary material for: Assessing kinesthetic proprioceptive function of the upper limb: a novel dynamic movement reproduction task using a robotic arm
Source: PeerJ. 2021 May 3;9:e11301. doi: 10.7717/peerj.11301 (PMC8101453; doi:10.7717/peerj.11301)
Supplement: Supplemental Information 5 [file peerj-09-11301-s005.zip › DMR scripts + variables/Variables.docx]

**Order of variables in raw data files:**

trial number; randomization trial number; condition; blindfold; direction; phase; mode; attempt; center x; center y; time; x position; z position; y position; radius; x force; z force; y force.

**Values:**

Trial number: 1-18

Note: trials 1-3 and 10-12 are practice trials; trials 4-9 and 13-18 are test trials

Randomization trial number: 1-18

Note: this variable is purely for programming purposes (to check randomization)

Condition: 1-2

Note: condition 1 = trials 1-9; condition 2 = trials 10-18

Blindfold: “blindfold”/“noBlindfold”

Direction: “clockwise”/“counterclockwise”

Phase: “practice”/“test”

Mode: “Guided”/“Free”

Note: guided = target movement; free = replication of target movement

Attempt: 1,2,3, …

Note: increase on movement in wrong direction during target (guided) movement

Center x: x coordinate (dm) of center of target (guided) circle

Center y: y coordinate (dm) of center of target (guided) circle

Time: time (s)

X position: x coordinate (dm)

Z position: z coordinate (dm)

Y position: y coordinate (dm)

Radius: distance between current position and center of target circle (dm)

X force: exerted force in x direction (N/m)

Z force: exerted force in z direction (N/m)

Y force: exerted force in y direction (N/m)
