# Supplementary material for: Assessing kinesthetic proprioceptive function of the upper limb: a novel dynamic movement reproduction task using a robotic arm
Source: PeerJ. 2021 May 3;9:e11301. doi: 10.7717/peerj.11301 (PMC8101453; doi:10.7717/peerj.11301)
Supplement: Supplemental Information 7 [file peerj-09-11301-s007.docx]

**Dynamic Movement Reproduction (DMR) directional error**

As described in the manuscript, the HapticMaster automatically logs position along all three dimensions every 2 ms during the DMR task. For each logged position, the distance between the middle point of the circular movement pattern and the current coordinates was calculated (i.e., reproduced radius). Subsequently, the target radius (i.e., 8 cm) was subtracted from this, and the absolute value served as the error (i.e., direction was not taken into account). Next, the mean of all errors was calculated per trial, and served as the DMR error, which is used to calculate DMR accuracy (Figure 4 in manuscript; see MATLAB file ‘HMData_mean_absolutedeviation_all’ in supplemental files for implementation). Note that the zero value of this measure means that there was absolutely no deviation (error) from the target circle movement pattern.

Here we introduce an additional measure that may be of interest to evaluate, DMR directional error, in which the difference between target radius (movement pattern) and reproduced radius (movement pattern) is used to calculate error, but the direction of this error (negative or positive) is taken into account. Such errors would allow us to determine if a participant is systematically overshooting or undershooting target movement during the DMR task (positive versus negative average DMR directional error, respectively), as is commonly reported in JR tasks. However, it is probable that most people will not systematically over- or under-shoot entire target movement patterns, but may vary in their performance errors throughout the movement pattern. This may create a situation where absolute DMR task error will be high (low accuracy), but DMR directional error may be low (high accuracy) because the errors ‘even out’. Here we explore whether the combination of DMR directional error, with or without visual inspection of the movement pattern data, is sufficient to delineate the type of movement pattern impairment (e.g., due to altered position sense, altered sensorimotor integration, or both).

During the DMR task, some participants may have impairments with sensorimotor integration, while position sense is intact. For example, a participant may be overshooting (reproduced radius > 8 cm) at some point, while undershooting at another (reproduced radius < 8 cm), but roughly placing the circle in the correct spatial position. Figure S1.1 shows an example of this as performed by one of the study participants. In this situation, absolute DMR error was relatively high (1.06 cm) compared to DMR directional error (0.02 cm), suggesting non-systematic over-shooting and undershooting errors.


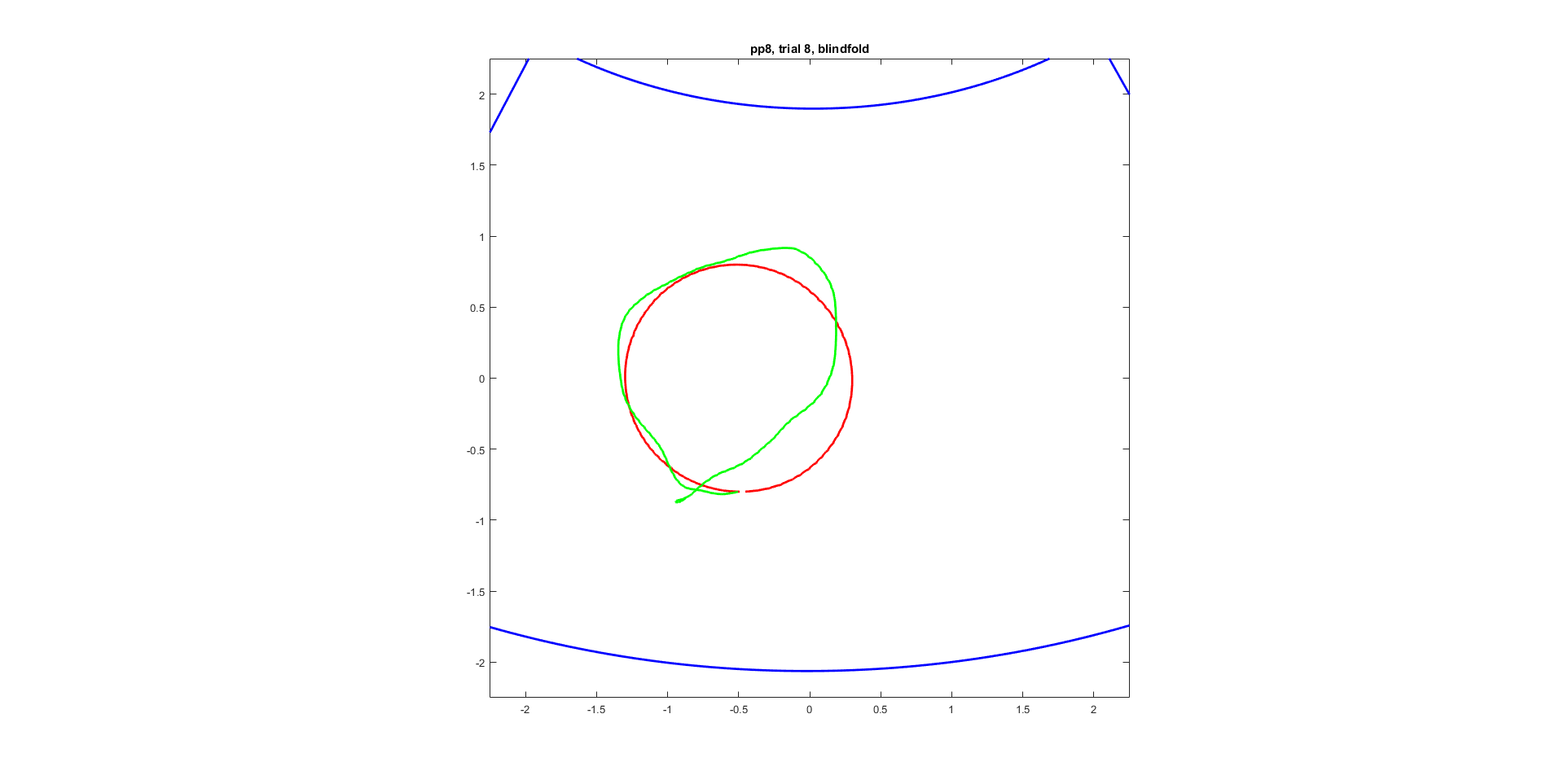


*Figure S1.1*. Visualization of raw data from a single test trial of the Dynamic Movement Reproduction task. Both the target (red line) and reproduced (green line) movement pattern are visualized. Note that position sense in this participant appears relatively intact, while the sensorimotor integration is relatively more impaired.

Alternatively, a participant’s sensorimotor integration may be relatively intact, while position sense is impaired. For example, a participant may be capable of performing a circular movement with the correct radius, though not in the correct position. Figure S1.2 shows an example of this as performed by one of the study participants. In this case, absolute DMR error (1.05 cm) was again relatively high compared to DMR directional error (0.04 cm).


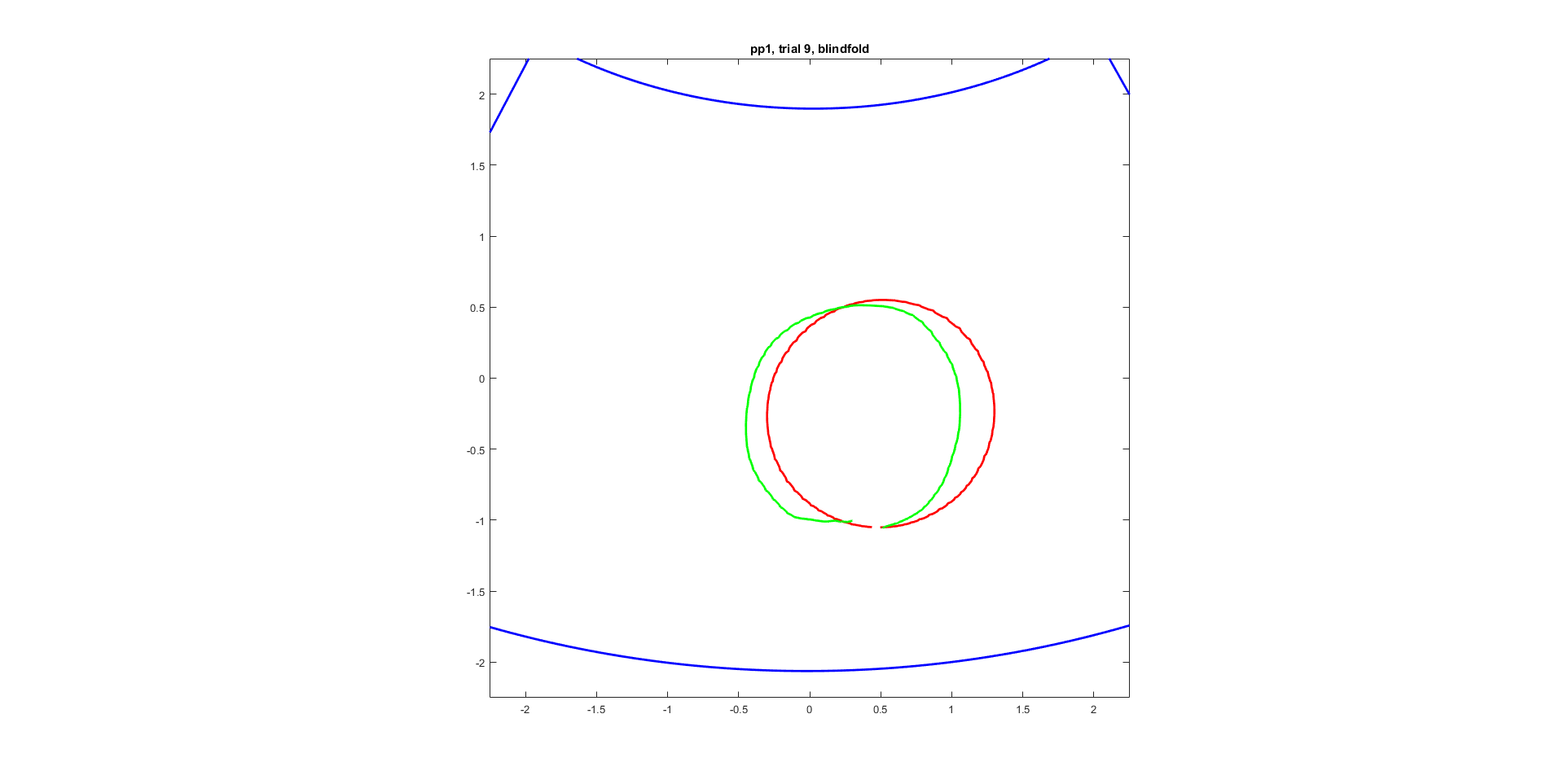


*Figure S1.2*. Visualization of raw data from a single test trial of the Dynamic Movement Reproduction task. Both the target (red line) and reproduced (green line) movement pattern are visualized. Note that sensorimotor integration in this participant appears relatively intact, while position sense is impaired.

Note that the DMR directional error measure mainly differs from absolute DMR error in the meaning of the zero value. While a value of zero for absolute DMR error indicates that *both* sensorimotor integration and position sense are intact, a value of zero for the DMR directional error indicates that there are no systematic errors, with *either* sensorimotor integration and/or position sense intact. That DMR directional error could be zero for either situation means that additional visual inspection of the data is necessary. Indeed, the two DMR measures are highly correlated (using No Visual Information data from Study 2 for sufficient statistical power: *ρ*= .68, *p*=<.001), suggesting that the DMR directional error does not add to data interpretation unless also accompanied by visual inspection of the data. Furthermore, similar to DMR accuracy, DMR directional accuracy (i.e., the average DMR directional error over 6 test trials) showed fair-to-good test-retest reliability (using No Visual Information test-retest data from Study 1: *ICC*=.73, *F*(22,22)=3.55, *p*=.002, *95%CI*=[.35-.88]) and a weak association with JR accuracy (using No Visual Information data from Study 2 for sufficient statistical power: *ρ*=.25, *p*=.04).
